# Vol. III, 11th Series, 1901

**Published:** 1903-03

**Authors:** 


Vol. III, 11th Series, 1901. These books are not reviews, but are clinical lectures from the best sources. The present volume is particularly interesting. Under therapeutics the following subjects are discussed: Phototherapy after Tinsen's methods; antitoxic serums, their preparation and standardization; clinical aspects of spa treatment; the influence of pregnancy on the prognosis and treatment of coexisting acute and chronic diseases; gonorrhea and marriage; on the drawbacks to the spinal use of cocaine and the accidents due to it; the selection of favorable cases of pulmonary tuberculosis for sanatorium treatment; remarks on the treatment of bleeders.

Under the headings of medicine, neurology, surgery, diseases of the eye, ear, nose and throat and laboratory methods the subjects discussed are equally interesting and important. T. J. B.

